# Characterization the complete chloroplast genome of *Euryale ferox* (Nymphaeaceae), an medicinal plant species in China

**DOI:** 10.1080/23802359.2020.1775516

**Published:** 2020-06-08

**Authors:** Zhiyong Guo, Jie Min

**Affiliations:** a The First Hospital of Nanchang, Jiangxi, China; b The Third Hospital of Nanchang, Jiangxi, China

**Keywords:** *Euryale ferox*, Nymphaeaceae, genetic evolution, chloroplast genome, medicinal plant

## Abstract

*Euryale ferox* belongs to the family Nymphaeaceae that has been used as foods and medicines in China. In this study, we had been completed the complete chloroplast genome of *Euryale ferox*, which is 159,930 bp in length and has four sub-regions. The complete chloroplast genome sequence of *Euryale ferox* contains 89,678 bp of a large single-copy region (LSC), 22,202 bp of small single-copy region (SSC) and 25,025 bp of two inverted repeat regions (IRs). The complete chloroplast genome of *Euryale ferox* is also consistent with other chloroplast genomes most plant species. The overall nucleotide composition of chloroplast genome sequence has: A (30.1%), T (30.8%), C (19.9%), G (19.2%) and the total GC content of 39.1%. Its sequence contains 127 genes, including 84 encoding genes, 35 transfer RNA genes and 8 ribosomal RNA genes. As the analysis result, the ML tree presents that *Euryale ferox* clustered with *Nuphar advena* belonging to the family Nymphaeaceae in genetic evolution relationship by the maximum likelihood (ML) methods.

*Euryale ferox* is an annual aquatic plant that is also the only species in the genus Euryale in the family Nymphaeaceae. The Seed of *E. ferox* is one of nutritious food and also used as the traditional Chinese medicine in China (Liu et al. 2018)*. Euryale ferox* (Qian-Shi in Chinese) as one of medicinal plant widely distributed in tropical and subtropical regions of east and Southeast Asia (Verma et al. 2010). The seed of *E. ferox* is rich in starch, proteins, vitamins, minerals and many other nutritional ingredients, which is also a significant component of contemporary the Traditional Chinese Medicine (TCM) and is used to treat many diseases, such as kidney failure, chronic diarrhea and so one (Das et al. 2006). In this study, we completed the complete chloroplast genome of *Euryale ferox*, which can be used to study the phylogenetic relationship of the family Nymphaeaceae, which also can be used for as well as for understanding evolutionary events of medicinal plant species for the future.

The fresh *Euryale ferox* was collected on the market of herb near the Third Hospital of Nanchang (28.66 N, 115.90E) that located at Nanchang, Jiangxi and China. The herbarium specimen of *E. ferox* (No. THNC-04) was stored at the Third Hospital of Nanchang. The total DNA was extracted from the fresh of *E. ferox* using the Plant Tissues Genomic DNA Extraction Kit (TIANGEN, BJ and CN) and was sequenced. The FastQC software (Andrews 2015) was used for purification the chloroplast genome DNA that used to control and remove the low quality sequences. The NOVOPlasty software (Dierckxsens et al. 2017) was assembled the chloroplast genome sequence of *E. ferox*. The Geneious software (Kearse et al. 2012) was used for annotation the chloroplast genome sequence of *E. ferox*. The CPGAVAS software (Liu et al. 2012) and the NCBI Blast search (https://blast.ncbi.nlm.nih.gov/Blast.cgi) were used to predict and correct all the genes on the chloroplast genome. At last, we submitted the annotated chloroplast genome sequence information of *E. ferox* to NCBI that the accession No. was NK9931121.

The complete chloroplast genome of *Euryale ferox* is 159,930 bp in length and has four sub-regions, which contains 89,678 bp of a large single-copy region (LSC), 22,202 bp of small single-copy region (SSC) and 25,025 bp of two inverted repeat regions (IRs). The complete chloroplast genome of *Euryale ferox* is also consistent with other chloroplast genomes most plant species. The overall nucleotide composition of chloroplast genome sequence has: A (30.1%), T (30.8%), C (19.9%), G (19.2%) and the total GC content of 39.1%. Its sequence contains 127 genes, including 84 encoding genes, 35 transfer RNA genes and 8 ribosomal RNA genes. The IR region harbors 15 genes, which has 5 protein-encoding genes, 6 tRNA genes and 4 rRNA genes.

Nine plant species the complete chloroplast genome sequences were used for constructing phylogenetic trees using maximum likelihood (bootstrap repeat is 2000) methods with the MAFFT software (Katoh and Standley 2013) and the MEGA X software (Kumar et al. 2018). The MEGA X software was used for constructing the ML phylogenetic trees and was inferred with strong support at all the nodes. At last, the MEGA X software was used for drawing and editing the ML tree, respectively. As the analysis result (Figure 1), the ML tree presents that *Euryale ferox* clustered with *Nuphar advena* belonging to the family Nymphaeaceae in genetic evolution relationship. This study result can be used to study the phylogenetic relationship of the family Nymphaeaceae, which also can be used for as well as for understanding evolutionary events of medicinal plant species.

**Figure 1.**
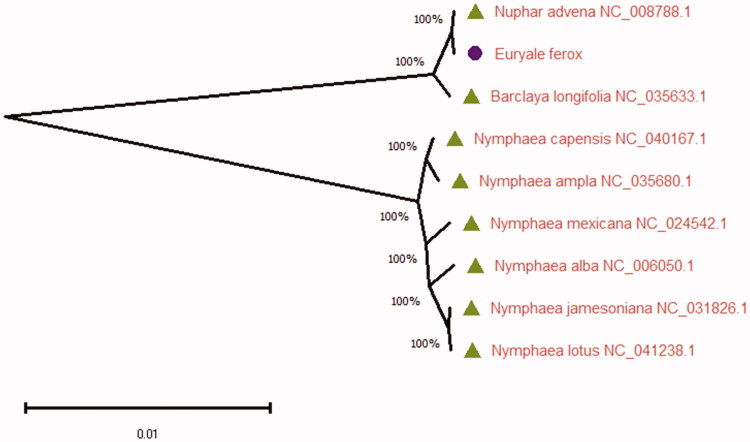
Maximum likelihood phylogenetic trees based on nine complete chloroplast genomes in this study. Bootstrap support values based on 2000 replicates are shown next to the nodes. The numbers above branches indicate bootstrap support values of maximum likelihood trees, respectively.

## Data Availability

The data that support the findings of this study are available from the corresponding author, upon reasonable request.

## Data openly available in a public repository that issues datasets with DOIs

The data that support the findings of this study are openly available in *Euryale ferox* at http://doi.org/[doi], reference number is NK9931121.
